# Switchable Charge Storage Mechanism via in Situ Activation of MXene Enables High Capacitance and Stability in Aqueous Electrolytes

**DOI:** 10.1021/acsnano.3c12226

**Published:** 2024-02-19

**Authors:** Cheng-Che Hsiao, James Kasten, Denis Johnson, Bright Ngozichukwu, Ray M. S. Yoo, Seungjoo Lee, Ali Erdemir, Abdoulaye Djire

**Affiliations:** † Artie McFerrin Department of Chemical Engineering, Texas A&M University, College Station, Texas 77843, United States; ‡ J. Mike Walker ’66 Department of Mechanical Engineering, Texas A&M University, College Station, Texas 77843, United States; § Department of Materials Science & Engineering, Texas A&M University, College Station, Texas 77843, United States

**Keywords:** MXene, Energy Storage, Intercalation, Electrochemical Activation, Aqueous

## Abstract

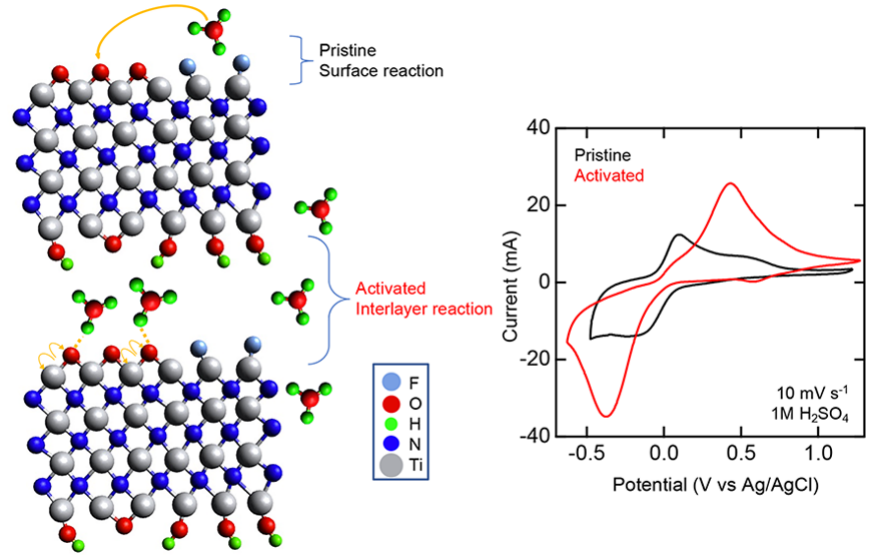

The need for reliable renewable energy storage devices has become increasingly important. However, the performance of current electrochemical energy storage devices is limited by either low energy or power densities and short lifespans. Herein, we report the synthesis and characterization of multilayer Ti_4_N_3_T_*x*_ MXene in various aqueous electrolytes. We demonstrate that Ti_4_N_3_T_*x*_ can be electrochemically activated through continuous cation intercalation over a 10 day period using cyclic voltammetry. A wide operating window of 2 V is maintained throughout activation. After activation, capacitance at 2 mV s^–1^ increases by 300%, 140%, and 500% in 1 M H_2_SO_4_, 1 M MgSO_4_, and 1 M KOH, respectively, while maintaining ∼600 F g^–1^ at 2 mV s^–1^ after 50000 cycles in 1 M H_2_SO_4_. This activation process is possibly attributed to the unique morphology of the multilayered material, allowing cation intercalation to increase access to redox-active sites between layers. This work adds to the growing repository of electrochemically stable MXenes reported for aqueous energy storage applications. These findings offer a reliable option for reliable energy storage devices with potential applications in large-scale grid storage and electric vehicles.

## 1 Introduction

Improving the performance of electrochemical energy storage devices is a necessary development for efficiently harvesting energy from renewable sources and gaining independence from a fossil-fuel-based energy economy.^1,2^ Currently, batteries and supercapacitors are at the forefront of electrochemical energy storage research due to their high energy and power densities, respectively.^3−5^ On one hand, lithium-ion batteries have emerged as an industry standard for various electrical energy storage applications due to their superior energy densities.^6^ However, their low power densities and short lifespans along with the fluctuations in lithium prices and environmental concerns have pushed investigations for cheaper and more environmentally benign alternative battery systems and materials with high energy and power densities.^7^

On the other hand, electrochemical double-layer capacitors (EDLCs) are limited to only providing high power and long cycle life.

Pseudocapacitors can provide a solution to these performance gaps by combining the best attributes from EDLCs and batteries. In the search to find cost-effective and efficient materials for supercapacitors, a wide range of materials including polymers,^8−11^ chalcogenides,^12−15^ metal oxides^16−20^ and sulfides^21−23^ and high surface area transition-metal carbides^24−27^ and nitrides^28−31^ have been studied. These materials are frequently investigated due to their pseudocapacitive charge storage mechanisms involving fast and reversible Faradaic redox reactions which contribute to substantially larger capacitances compared to EDLCs.^32^ Current benchmark materials for supercapacitors include RuO_2_, MnO_2_, and high surface area VN.^33−35^ However, transition metal oxides are known for exhibiting poor electrical conductivity, requiring them to be engineered at nanoscales to achieve pseudocapacitive kinetics, and thereby limiting their application to small-scale electronics.

An emerging class of 2D transition metal carbides and nitrides known as MXenes was developed in 2011 by etching of a precursor M_*n*+1_AX_*n*_ phase, where M represents an early d-block transition metal, A represents a group IIIA–VIA element, and X represents carbon and/or nitrogen.^36^ The A layer can be selectively etched to produce a MXene with structure M_*n*+1_X_*n*_T_*x*_, where T_*x*_ represents surface termination groups (−O–, −OH, −F, etc.). Currently, approximately 100 MXene compositions have been reported to theoretically exist, with most of them experimentally synthesized.^37^ Their morphology, high conductivity, and active surface area have made them applicable materials for battery and supercapacitor research.^38^ However, between carbide and nitride MXenes, the former has received far more attention, as there are a greater number of possible carbide compositions. Additionally, is has been reported to be significantly harder to synthesize nitride MXenes due to the high formation energy of their precursor MAX phases.^39^ Nonetheless, nitride MXenes have been reported to possess greater conductivity, oxidative stability, and active surface area compared to their carbide counterparts.^40^ Both carbide and nitride MXenes have shown great potential for aqueous supercapacitors with Ti_3_C_2_, V_2_C, V_4_C_3_, and Ti_2_N each obtaining high capacitances of over 200 F g^–1^ in select aqueous electrolytes, along with V_2_C and Ti_2_N also obtaining stable capacitance retentions.^41−43^ However, no MXene to date has exhibited high stability across aqueous electrolytes of different pHs, but rather selective stability in one electrolyte. Similarly, no MXene to date has been reported to possess capacitance growth in more than one aqueous environment.

In this work, we report on the electrochemical performance, in aqueous electrolytes, of multilayered Ti_4_N_3_T_*x*_ MXene synthesized via an oxygen-assisted molten salt etching to remove the aluminum layer of Ti_4_AlN_3_. MXene synthesis was verified using multiple physical characterization analyses, including X-ray diffraction (XRD), scanning electron microscopy (SEM), and Raman spectroscopy. The surface termination groups (T_*x*_) were also characterized via Fourier transform infrared (FTIR) spectroscopy. After physical characterization, we electrochemically activated multilayered Ti_4_N_3_T_*x*_ through continuous cation intercalation over a 10 day period using cyclic voltammetry (CV). The electrochemical performance and capacitance of Ti_4_N_3_T_*x*_ were assessed before and after the activation process by using CV, electrochemical impedance spectroscopy (EIS), and galvanostatic charge–discharge (GCD) in separate 1 M aqueous solutions of H_2_SO_4_, MgSO_4_, and KOH. Lastly, physical characterization was repeated after activation to investigate potential changes in material properties. In H_2_SO_4_, activation led to a switch in the charge storage mechanism from a capacitor to a capacitor–battery hybrid behavior as a result of hydronium ion intercalation, accompanied by changes in the oxidation state of Ti. Using these results, a proposed pseudocapacitive mechanism of Ti_4_N_3_T_*x*_ in H_2_SO_4_ was determined, which can be used to warrant further understanding of nitride MXene charge storage mechanisms for energy storage applications.

## 2 Results and Discussion

### 2.1 Physical Characterization of Ti_4_N_3_T_*x*_

#### 2.1.1 Physical Characterization

**Figure 1:**
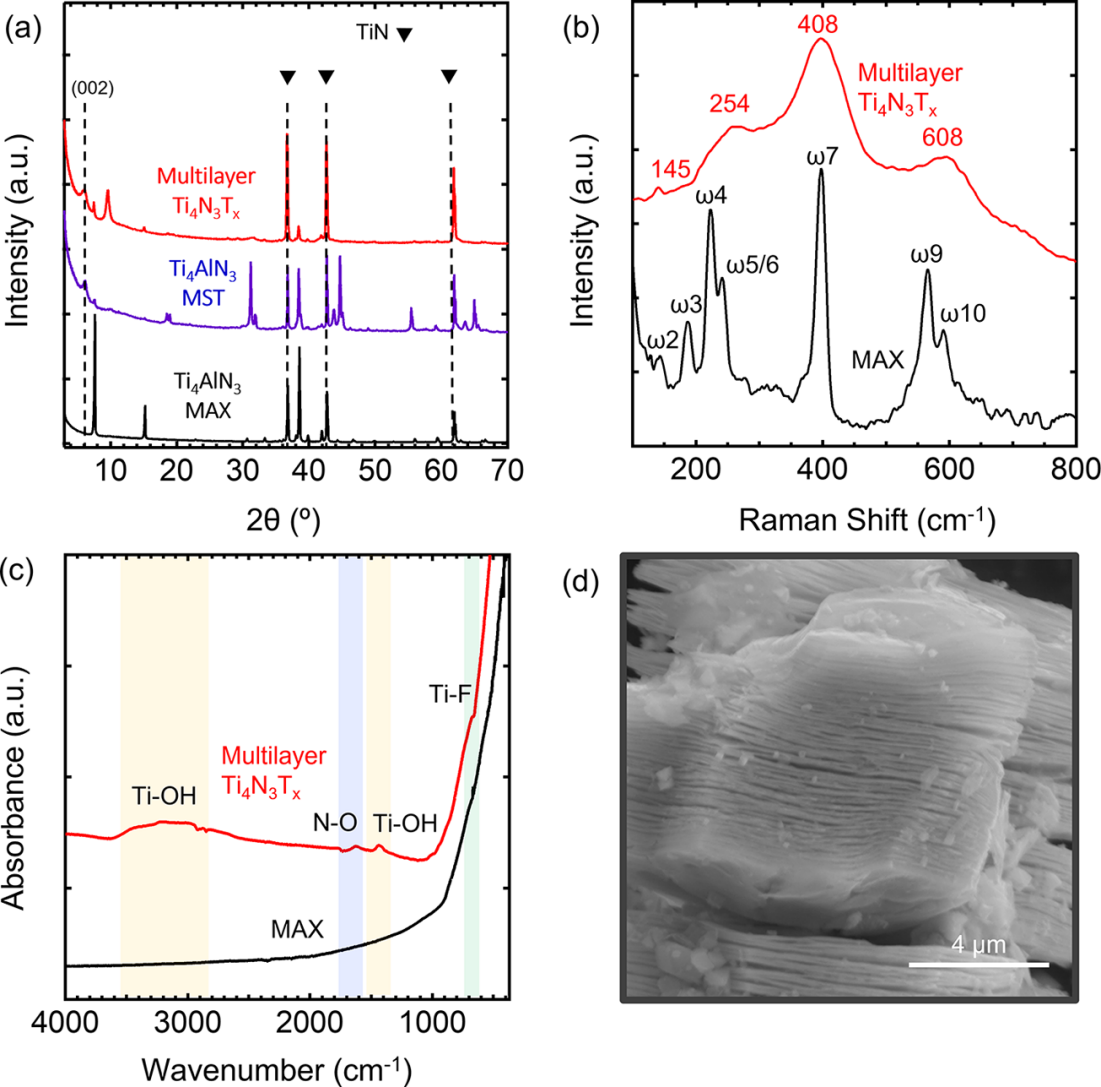
(a) X-ray diffraction patterns of Ti_4_AlN_3_ MAX phase (black), Ti_4_AlN_3_ molten salt fluoride treated (MST) (purple) from the O_2_-assisted molten salt fluoride synthesis, and multilayer Ti_4_N_3_T_*x*_ MXene (red). (b) Raman and (c) FTIR spectra of MAX (black) and MXene (red). (d) SEM image for multilayer Ti_4_N_3_T_*x*_ MXene.

The XRD patterns of the Ti_4_AlN_3_ MAX phase precursor, molten salt treated Ti_4_AlN_3_-MST, and multilayered (ML) Ti_4_N_3_T_*x*_ MXene are shown in Figure 1a. The synthesis is corroborated by a shift in the (002) diffraction peak toward a lower angle from 2θ = 7.56° to 5.92°, which indicates sufficient etching of the Al layer from Ti_4_AlN_3_ to ML Ti_4_N_3_T_*x*_. This shift is accompanied by an increase in the *c*-lattice parameter (*c-LP*) from 23.4 Å for the Ti_4_AlN_3_ MAX phase to 29.8 Å for the multilayer Ti_4_N_3_T_*x*_, which is consistent with previously reported values.^44−46^ Furthermore, most of the peaks belonging to Ti_4_AlN_3_ either are absent or have significantly decreased in intensity following etching. All the peaks have been identified except that at 2θ = 10.17°. This unknown peak seems to appear after the acid wash step, suggesting that it may be related to the interaction of the acid solution and the fluoride salts. Further studies are needed to understand the nature of this peak. Extra peaks in the Ti_4_N_3_-MST spectra that are not present in the MAX phase are the expected aluminum fluoride compounds which include K_2_NaAlF_6_, K_2_Li[AlF_6_], Na_3_AlF_6_, K_2_Na[AlF_4_]_3_, and Na_3_AlF_6_, all of which are soluble in the formic acid solution.^44^ Also, additional peaks present in the multilayer MXene at 2θ = 38.61, 44.79, and 65.18° are attributed to unreacted TiN which was originally present in the MAX phase.^44,47^ The physical surface area was investigated by N_2_-physisorption. The Ti_4_AlN_3_ MAX phase shows a surface area of 2.5 m^2^ g^–1^ while the multilayer Ti_4_N_3_T_*x*_ MXene displays an increase in the surface area to 18 m^2^ g^–1^. An increase in pore diameter and pore size (Figure S1) with an increase in pore volume from about 0.007 cm^3^ g^–1^ in the MAX to 0.05 cm^3^ g^–1^ in the Ti_4_N_3_T_*x*_ MXene is related to the pores generated from voids between the multilayer sheets.^44^ The Raman spectrum of the Ti_4_AlN_3_ MAX phase (Figure 1b) is shown to be consistent with previously reported data.^48^ In particular, ω_2_, ω_5_, and ω_10_ are E_1g_ group vibrations, which contain in-plane vibrational modes of Ti and N atoms.^49^ After etching the Al layer, these peaks decrease and broaden due to the increased interlayer spacing of the MXene structure. Similarly, ω_4_, ω_7_, and ω_8_ corresponding to A_1g_ out of plane vibrations of Ti and N atoms undergoing a red shift and broadening after the removal of the Al atom. Based on the group theory of M_4_X_3_ MXenes, there should be even more vibrational modes observed, as they involve more atomic layers and thus more possibilities of vibrational modes. The reason for the Ti_2_NT_*x*_ and Ti_4_N_3_T_*x*_ MXenes having the same Raman spectra is to be investigated and determined in future works. FTIR spectroscopy was used to identify the surface termination groups of the synthesized multilayer Ti_4_N_3_T_*x*_ MXene (Figure 1c). The broad and predominant peaks that emerge at 3300 and 1400 cm^–1^ are assigned to the vibrational stretching and bending of the OH group. The peak at 1600 cm^–1^ indicates N–H bonding resulting from the sublattice N atoms being exposed during acid washing.^50^ SEM was employed to investigate the morphology, completion of the etching process, and structural defects of both the Ti_4_AlN_3_ MAX (Figure S2a) and the multilayer Ti_4_N_3_T_*x*_ MXene (Figure 1d). The morphology of the MAX shows that the titanium nitride layers are held firmly together by the aluminum. However, the SEM of the MXene reveals a large interlayer distance between each flake, confirming etching of the Al layers from the parent MAX.^51^ Compositional analysis of the MAX and the MXene was performed using energy-dispersive X-ray spectroscopy (EDS) to gain insight into the atomic ratio of the Ti, Al, and N elements. Results confirm the removal of the Al with a negligible amount left compared to the MAX. The various atomic ratios are given in Figure S2. Residual K still present in the MXene arises from the molten salt fluoride used during the etching process.

#### 2.1.2 X-ray Absorption Spectroscopy

**Figure 2:**
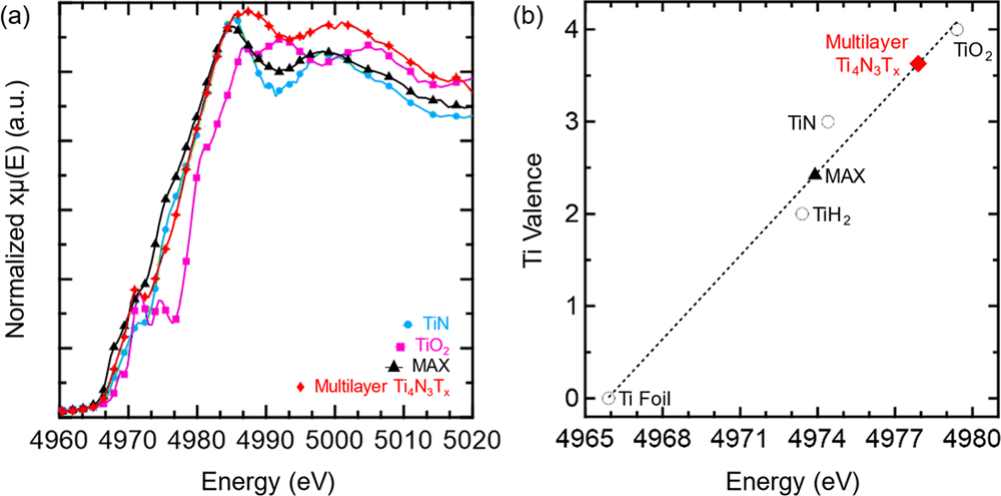
(a) XANES region of the normalized Ti K-edge XAS spectra for TiN (blue), TiO_2_ (magenta), Ti_4_AlN_3_ MAX (black), and multilayer Ti_4_N_3_T_*x*_ (red) materials. TiN and TiO_2_ are used as calibration standards. (b) Edge position determined from XANES spectra of several Ti reference compounds (hollow circles), Ti_4_AlN_3_ MAX (black triangle), and multilayer Ti_4_N_3_T_*x*_ (red diamond) as a function of Ti valency.

X-ray absorption spectroscopy (XAS) was performed to gather insight into how material oxidation state shifts during synthesis. Through analysis of the X-ray absorption near edge structure (XANES) region (Figure 2a), it can be observed that the MAX phase (black trace) lies to the left of the TiN (blue) reference curve. Upon etching, the Ti K-edge peaks of MXene (red) shift to the right to lie between the TiN and TiO_2_ (purple) reference samples. This is further corroboration of the etching of the Al layers and integration of oxygen surface termination groups.^52,53^ The XANES data were further investigated to ascertain the valency of the Ti atoms in the MAX and MXene structures. Based on the calibration curve generated by taking the derivative of the reference curves and finding peak energy values (Figure 2b), a calibration curve can be generated with an *R*^2^ value of 0.97, indicating high reliability of the curve. The calculated reference values are also in line with values from the literature, indicating high accuracy for further analysis.^54,55^ From this calibration curve, the Ti_4_AlN_3_ MAX phase has a Ti valency of 2.4, while the multilayer Ti_4_N_3_T_*x*_ MXene has a Ti valency of 3.6. The deviation from the expected +3 valency can be attributed to the other bonds being made by the Ti atoms in the structure. Lower Ti oxidation states in the MAX are attributed to metal–metal Ti–Al bonds to form the 3D cross-links. Meanwhile, in the MXene structure, Ti atoms simultaneously participate in a combination of +3 Ti–N bonds and multiple +2 bonds with termination groups to a single Ti atom.

### 2.2 Electrochemical Results

The intercalation of cations, such as Li^+^, Na^+^, Mg^2+^, and K^+^, has been demonstrated for carbide MXenes, but no interaction chemistry has been extensively reported for nitride MXenes. For Ti_3_C_2_T_*x*_, this intercalation has led to high pseudocapacitance with adequate stability, especially in aqueous electrolytes.^41^ Here, we use this intercalation chemistry to activate nitride MXene electrodes in acidic, basic, and neutral aqueous electrolytes over wide voltage windows and to increase capacitance over time. We use 1 M solutions of H_2_SO_4_, MgSO_4_, and KOH to represent the different pH regimes—acid, neutral, and base. To activate the material, we cycled a fresh electrode in each of the electrolytes using CV at a scan rate of 50 mV s^–1^. The activation consists of intercalating H_3_O^+^, Mg^2+^, and K^+^ into the layers of Ti_4_N_3_T_*x*_ (T_*x*_ = O, OH, and F) and oxidizing and reducing the inner layer Ti, which are otherwise not accessible during conventional charge storage. To achieve full activation, the CV cycling was continued for 10 days in each electrolyte. After full activation, the capacitance is expected to increase.

#### 2.2.1 Electrochemical Intercalation and Capacitance Evolution

**Figure 3:**
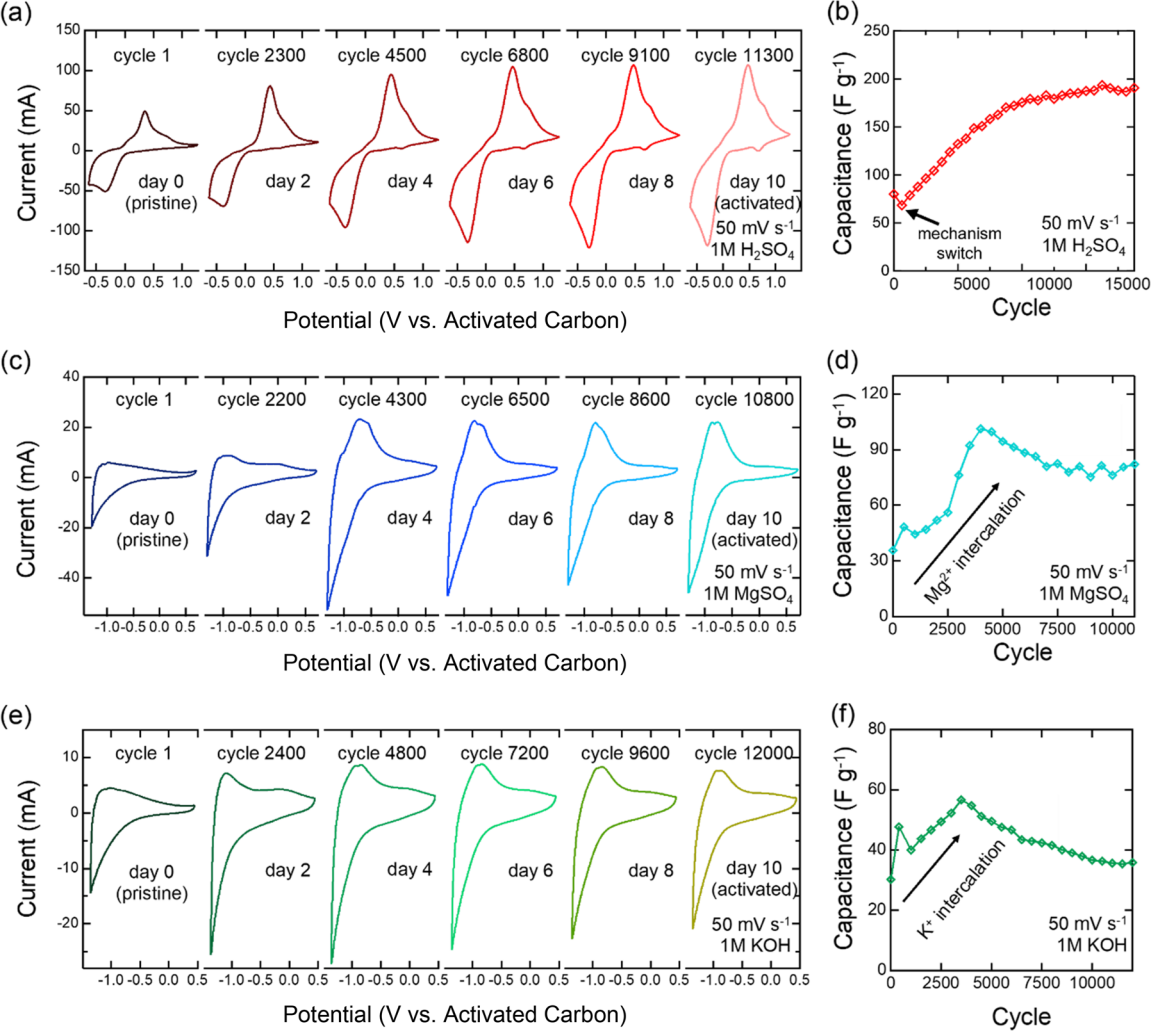
CV and specific capacitance evolution of multilayered Ti_4_N_3_T_*x*_ electrode subjected to continuous cycling over a 10 day period in (a, b) 1 M H_2_SO_4_, (c, d) 1 M MgSO_4_, and (e, f) 1 M KOH. CV measurements were taken at a 50 mV s^–1^ scan rate.

After material synthesis and electrode preparation, electrochemical measurements were conducted in aqueous 1 M H_2_SO_4_, MgSO_4_, and KOH electrolytes. Each electrode was tested in a fresh electrolyte and was electrochemically activated. Cyclic voltammetry (CV) scans reveal a wide voltage window of 1.9 V in 1 M H_2_SO_4_ (Figure 3a) electrolyte. As shown in Figure 3b, capacitance increases from ∼70 F g^–1^ to ∼190 F g^–1^ at a 50 mV s^–1^ scan rate, representing a capacitance retention of 270% over the 10 day period of continuous cycling. The redox peaks seen in Figure 3a may be attributed to a quasi-reversible protonation between aqueous hydronium and −O– surface termination groups present between MXene layers, which is discussed later. The growth in these peaks during cycling indicates improved Faradaic charge-storage behavior between layers, which is consistent with the capacitance results in Figure 3b.

In alkaline and neutral systems, Ti_4_N_3_T_*x*_ MXene exhibits working voltage windows of 2.0 V in MgSO_4_ (Figure 3c) and 1.8 V in KOH (Figure 3e). Pseudocapacitive activity can be observed in the cathodic region of both neutral and basic electrolytes due to the intercalation of cations (K^+^, Mg^2+^).^41^ Moreover, the CV shape in MgSO_4_ appears similar to that in previous nitride MXene works, demonstrating pseudocapacitive behavior.^56^ It is worth noting that hydrogen evolution becomes more pronounced during activation. Moreover, the capacitance reached its maximum between cycle 3000 and 5000 (Figure 3d), with over 100 F g^–1^ in MgSO_4_ and about 60 F g^–1^ in KOH electrolyte at 50 mV s^–1^. After reaching the maximum, the capacitance then stabilizes. In MgSO_4_ and KOH, the capacitance retentions are about 220% and 125%, respectively, at the end of the 10 day activation period.

#### 2.2.2 Capacitance Comparison between Pristine and Activated Electrodes

**Figure 4:**
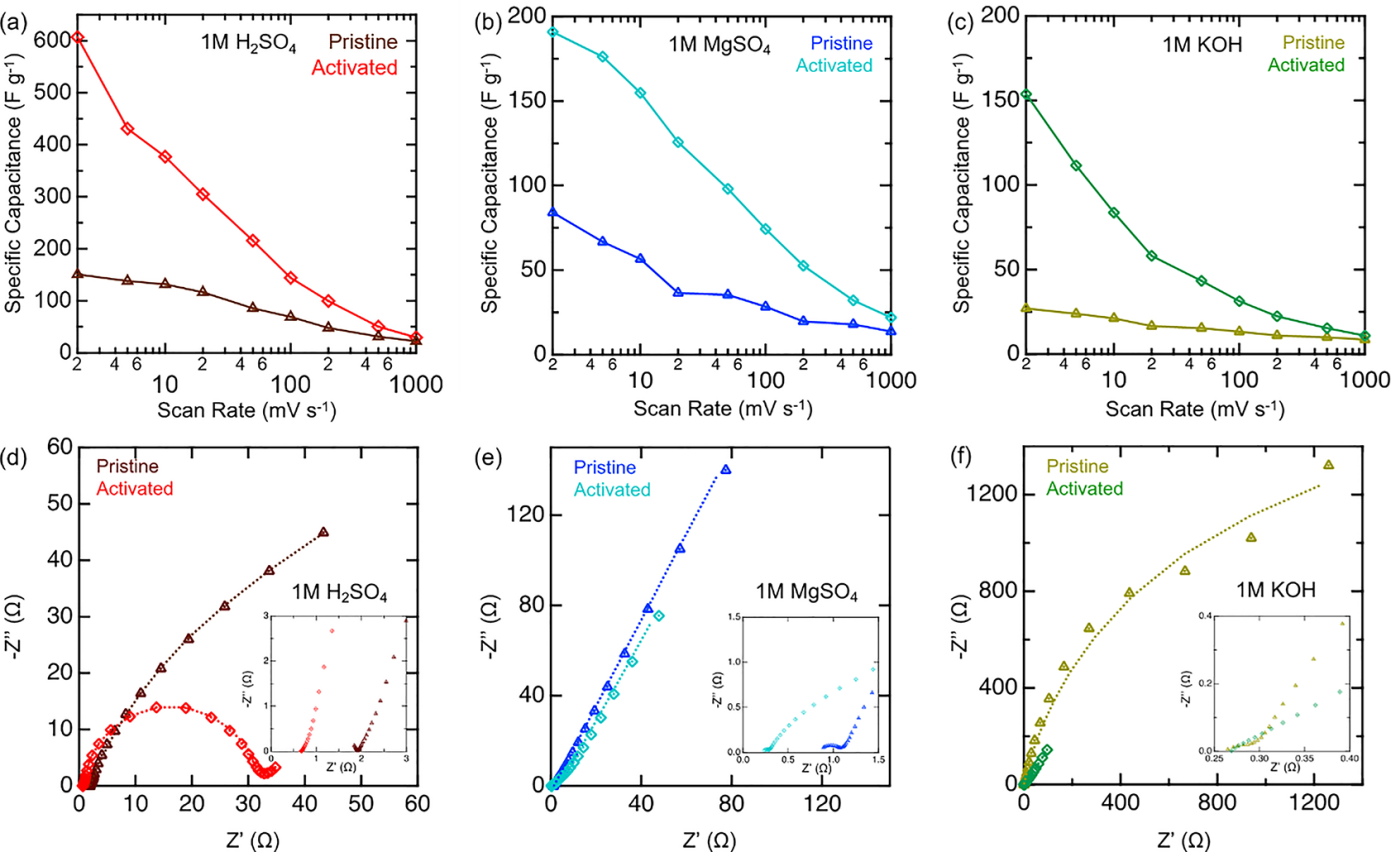
Comparison of electrochemical behavior of pristine (triangle) and activated (diamond) multilayered Ti_4_N_3_T_*x*_ electrodes in (a, d) 1 M H_2_SO_4_, (b, e) 1 M MgSO_4_, and (c, f) 1 M KOH electrolyte. (a–c) Specific capacitance of Ti_4_N_3_T_*x*_ as a function of scan rate. (d–f) Nyquist plots of the Ti_4_N_3_T_*x*_ electrodes including the circuit fitting.

After the working voltage window was determined, CV at scan rates from 2 to 1000 mV s^–1^ were taken for pristine and activated Ti_4_N_3_T_*x*_. The gravimetric capacitances were then calculated for H_2_SO_4_, MgSO_4_, and KOH based on the active material mass loading (Figure 4a–c). Activated electrodes exhibited capacitances of over 600 F g^–1^ in H_2_SO_4_, 190 F g^–1^ in MgSO_4_, and 150 F g^–1^ in KOH electrolyte. Moreover, the capacitance values at 2 mV s^–1^ increased after activation by 300% in H_2_SO_4_, 140% in MgSO_4_, and 500 in KOH. The increase in capacitance of the electrodes is likely due to the intercalation of cations between the MXene layers. Further analysis of the activation mechanism will be investigated in future works.

#### 2.2.3 Electrochemical Impedance Spectroscopy

Electrochemical impedance spectroscopy (EIS) was used before and after activation in each electrolyte to gain insight on the processes occurring at the electrode–electrolyte interface. All Nyquist plots were collected centered at open circuit potential (OCP). Prior to activation, the EIS spectrum in H_2_SO_4_ (Figure 4d) revealed a very fast surface-controlled double-layer process followed by an ion diffusion process, as evidenced by an inconspicuous semicircle followed by an inclined line in the spectrum. However, after activation in H_2_SO_4_, the electrode’s spectrum switches to a large semicircle, characteristic of a much slower charge transfer step followed by an inclined line for the ion diffusion process. This “switch” in the charge storage mechanism is likely the result of the hydronium ions successively intercalating between the layers of the multilayered Ti_4_N_3_T_*x*_, followed by protonation and deprotonation of −O– termination groups. This redox process is evidenced by the redox couple in the CV. However, this “switch” phenomenon was not observed in the EIS spectra of MgSO_4_ (Figure 4e) and KOH (Figure 4f) electrolytes, which is also consistent with the CV results, where a rapid non-Faradaic process in the high-frequency region followed by an ion diffusion process in the low-frequency region was observed in each. The EIS spectra show consistent capacitive behavior for both pristine and activated material. Interestingly, the equivalent series resistance (ESR) in H_2_SO_4_ and MgSO_4_ systems was reduced via the activation process but remained about the same in KOH.

#### 2.2.4 Galvanostatic Charge–Discharge

**Figure 5:**
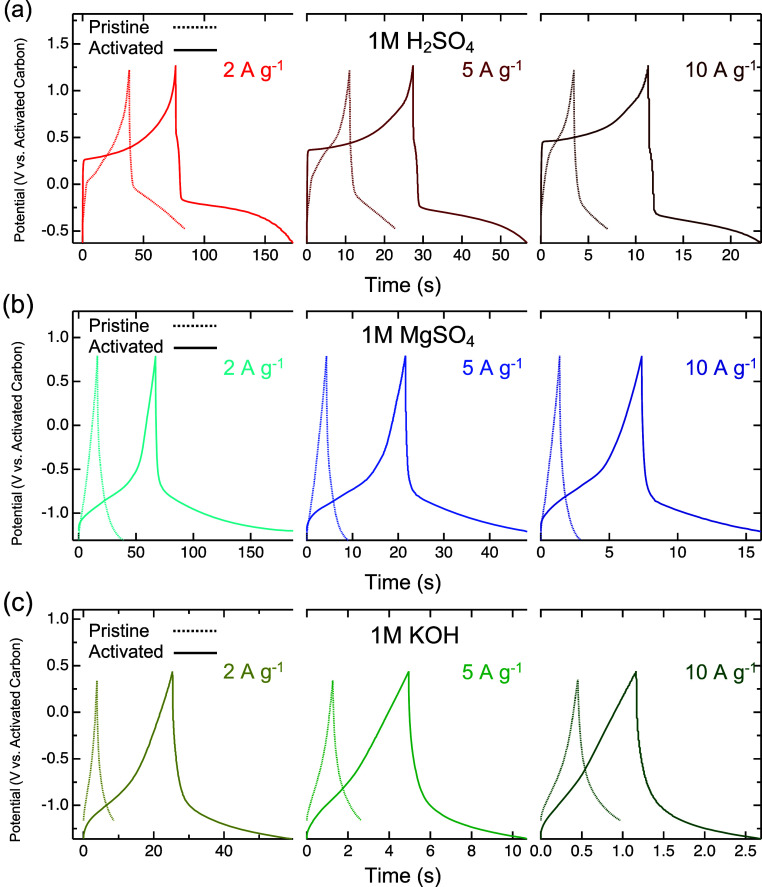
Galvanostatic charge–discharge curves of multilayered Ti_4_N_3_T_*x*_ electrodes at different current densities in (a) 1 M H_2_SO_4_, (b) 1 M MgSO_4_ and (c) 1 M KOH. The dashed and solid lines represent the pristine and activated electrode, respectively. The charge–discharge curves are consistent with the different current densities.

Galvanostatic charge–discharge (GCD) curves were taken at varying charge/discharge rates from 2 to 100 A g^–1^ to further investigate the charge storage mechanism and the energy storage performance before and after activation. Following activation, the charge/discharge times increased in each electrolyte, indicating a higher capacity. Like EIS, GCD results suggest a “switch” in the charge storage mechanism in the H_2_SO_4_ activated Ti_4_N_3_T_*x*_. The discharge curve after activation in H_2_SO_4_ displays a mixed capacitive and battery behavior, as evidenced by a sharp voltage drop followed by a plateau (Figure 5a). The sharp drop indicates the rapid double-layer capacitive discharge, while the plateau represents the slower diffusion of ions between the layers of the multilayered Ti_4_N_3_T_*x*_. Meanwhile, only pseudocapacitive behavior was observed in the discharge curves for the MgSO_4_ and KOH electrolytes (Figure 5b,c). After activation, capacities of 50, 65, and 17 mAh g^–1^ were exhibited in H_2_SO_4_, MgSO_4_, and KOH electrolytes, respectively. This corresponds to increases of 100% in H_2_SO_4_, 400% in MgSO_4_, and 600% in KOH at 2 A g^–1^ (Figures S5–S7).

#### 2.2.5 Charge Storage Kinetics

**Figure 6:**
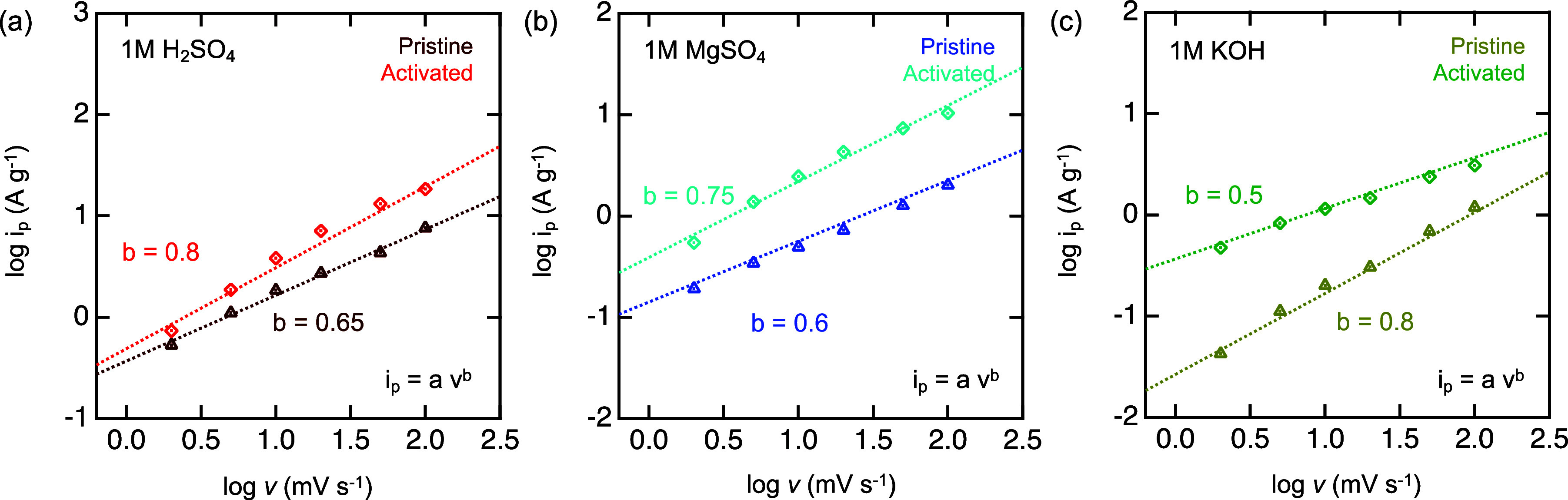
Scan rate dependence of the current for multilayered Ti_4_N_3_T_*x*_ in (a) H_2_SO_4_ (brown, red), (b) MgSO_4_ (navy, light blue), and (c) KOH (mustard, green) electrolytes before (triangles) and after (diamonds) the activation. The dashed line is the linear fit of each data set.

The charge storage kinetics were studied by analyzing the scan rate dependence of the peak current (Figure 6), using the equation^57^
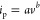
1where *i*_p_ is the gravimetric current in A g^–1^, v is the scan rate in mV s^–1^, and *a* and *b* are fitting parameters. The *b* value is utilized to obtain insights into the charge storage kinetics. For example, a *b* value of 1 represents capacitive storage with fast diffusion, whereas a value of 0.5 indicates diffusion-controlled processes. In H_2_SO_4_ (Figure 6a), during activation, the *b* value increases from 0.65 to 0.8, indicating charge storage is less limited by diffusion after activation. These kinetics are representative of previous reports studying pure MXene electrodes for aqueous supercapacitors.^42^ This phenomenon may be attributed to hydronium ions already being intercalated between the layers of MXene. In neutral electrolyte (Figure 6b), a similar phenomenon occurs wherein capacitive behavior and diffusion-controlled processes contribute to the kinetics of the activated electrode. In alkaline electrolyte (Figure 6c), however, the *b* value decreases from 0.8 to 0.5, indicating the kinetics is limited by ion diffusion. This is further reflected by the steady decrease in capacitance following activation before stabilization and may be attributed to the solution pH. Further studies involving more invasive techniques, such as in situ XRD and EQCM, being conducted during activation will reveal more about the electrolyte effect on kinetics over time.

#### 2.2.6 Long-Term Stability

**Figure 7:**
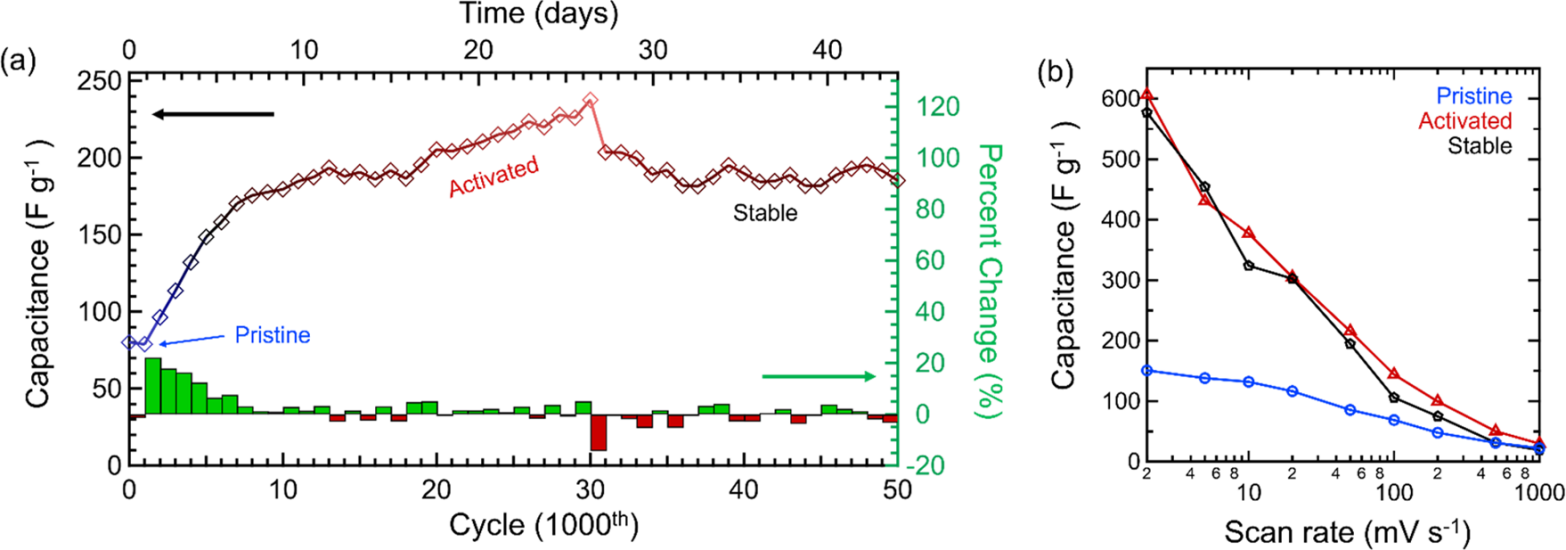
CV stability of multilayered Ti_4_N_3_T_*x*_ in 1 M H_2_SO_4_ at 50 mV s^–1^ over 50000 cycles (44 days). (a) Specific capacitance and percent change across CV cycling. (b) Specific capacitance as a function of scan rate at pristine (blue), activated (red), and stable (black) regions.

To evaluate long-term performance, in addition to activation, CV was continuously run in 1 M H_2_SO_4_ totalling 50000 cycles over the course of 44 days (Figure 7a). Following activation, capacitance stabilizes at ∼190 F g^–1^ for 5000 cycles before gradually increasing to a maximum capacitance of 237 F g^–1^ at 30000 cycles. After 30000 cycles capaticance consistently restabilizes to 190 ± 5 F g^–1^ for the remaining 20000 cycles. Although performance appears to decrease after 30000 cycles, capacitance across scan rate is maintained from cycle 15000 to cycle 50000 (Figure 7b), reaching over 575 F g^–1^ at 2 mV s^–1^. There have been many reports on as-synthesized MXenes evaluated as aqueous supercapacitor electrodes, but very few exhibit long-term capacitance retentions >100% and across multiple systems as shown in this report (Table S1).^41,42,56,58−61^ Recently, shear delamination has been effective at producing Ti_3_C_2_ sheets with cycle lives of up to 500000 CV cycles at a capacitance retention of ∼96%.^62^ However, to date, no other work has observed an increase and stabilization of as-synthesized multilayered MXene for the time scale observed in this work.

### 2.3 Post Characterization

#### 2.3.1 Fourier Transform Infrared Spectroscopy

**Figure 8:**
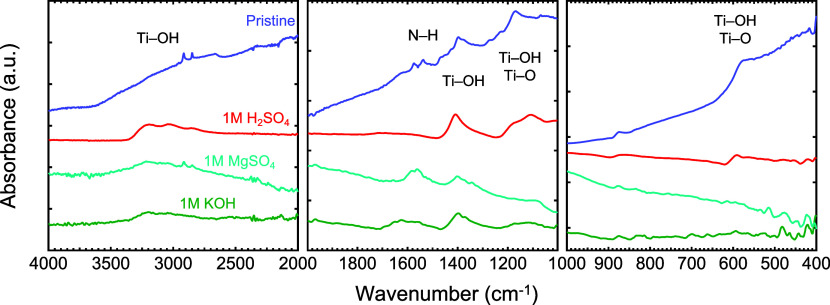
FTIR spectra of the multilayered Ti_4_N_3_T_*x*_ MXene electrodes before (dark blue) and after activation in 1 M H_2_SO_4_ (red), MgSO_4_ (light blue), and KOH (green).

To understand the effect of activation on the surface termination groups of MXene, FTIR spectroscopy was performed before and after activation in H_2_SO_4_, MgSO_4_, and KOH electrolytes (Figure 8). For all pristine and activated samples, the FTIR analysis revealed the presence of characteristic peaks at ∼3300 and ∼1400 cm^–1^, which are assigned to the stretching and bending vibrations of the −OH group, arising from the strong adsorption and coordination of water molecules on the electrode surface. It is well-known that the hydroxyl groups can act as active sites for electrochemical reactions, ultimately leading to an improvement in energy storage.^63^ A peak at ∼1550 cm^–1^, arising from the vibrational N–H stretching bonds, is present in both the KOH and MgSO_4_ electrodes, while being absent in the H_2_SO_4_ sample. A plausible explanation for the absence of this peak in the H_2_SO_4_ sample could be the presence of excess protons in the electrolyte allowing for the reduction of the N–H bond. In addition, a broad peak centered between 500 and 600 cm^–1^ was detected in the pristine and activated electrode in H_2_SO_4_ electrolyte but was absent in both KOH and MgSO_4_. These peaks are indicative of Ti–O and Ti–OH surface groups, respectively. Furthermore, the predominantly broad vibrational peak of Ti–O and Ti–OH groups at ∼1100 cm^–1^ was observed in all electrodes.^64^

#### 2.3.2 Raman Spectroscopy

**Figure 9:**
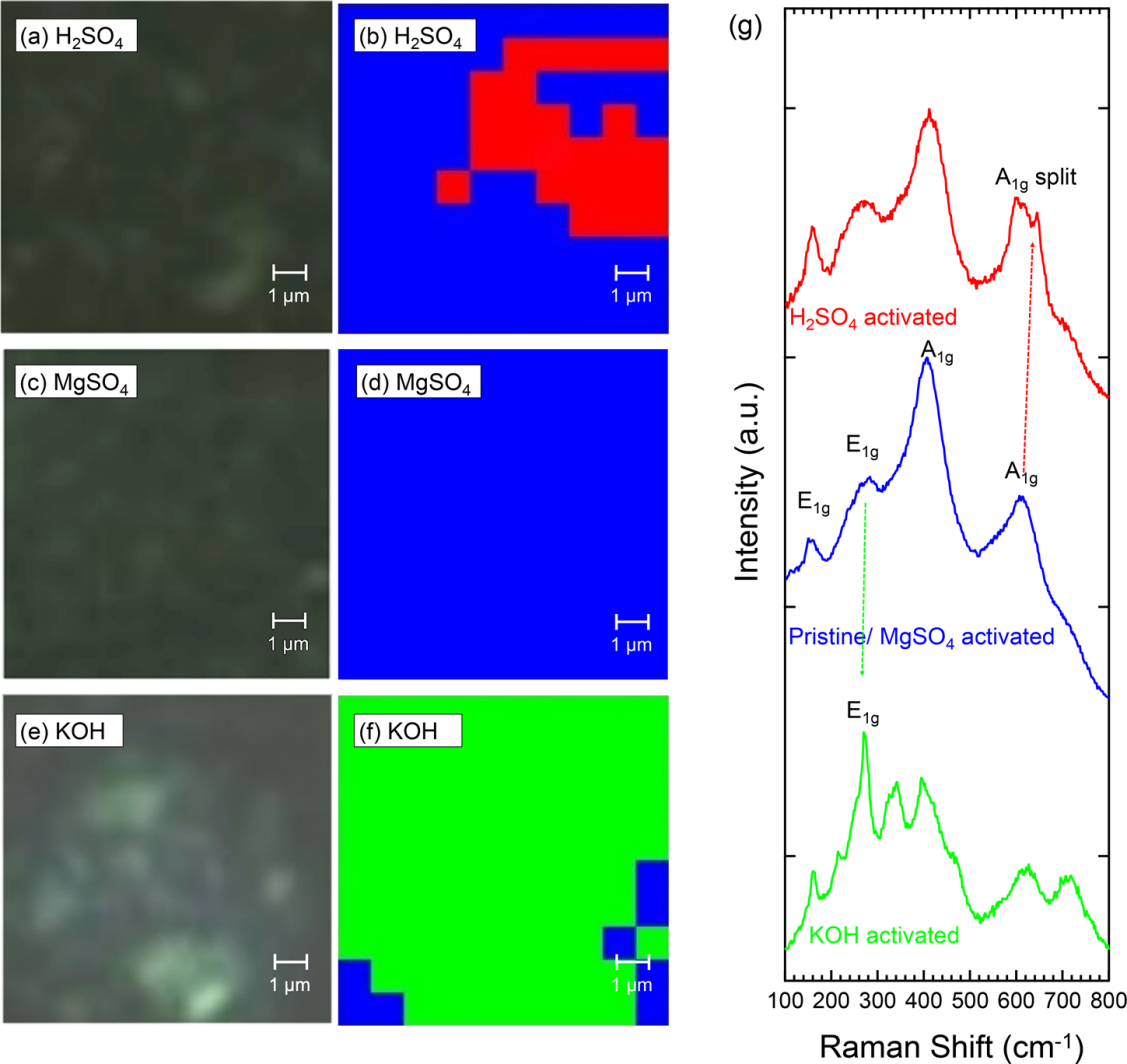
Raman mapping and the corresponding white light image of activated Ti_4_N_3_T_*x*_ MXene after electrochemical characterization in (a, b) H_2_SO_4_, (c, d) MgSO_4_, and (e, f) KOH. The blue spots indicate that the gathered spectrum is consistent with the pristine electrode. The red spots indicate the presence of splitting of the A_1g_ vibrational mode in the Ti_4_N_3_T_*x*_ MXene spectrum. The green spots indicate where modification of the E_1g_ vibrational mode at 426 cm^–1^ occurs. (g) Raman spectra of pristine and activated Ti_4_N_3_T_*x*_ MXene. All spectra were collected using a 532 nm laser, with 100% laser power, 10 s exposure time, 1800 lines/mm grating, and 100× objective lens.

To analyze and visualize the structural changes arising from the electrochemical processes, Raman mapping of the activated Ti_4_N_3_T_*x*_ electrodes was conducted. Due to the greatest change in potential mechanism, analysis of the H_2_SO_4_-activated Ti_4_N_3_T_*x*_ was conducted first (Figure 9a,b). The mapped spectrum displays the pristine (blue) and modified (red) areas of the material, based on the two Raman spectra observed in this region (Figure 9g, blue and red traces, respectively). Specifically, with the material activated under the H_2_SO_4_ electrolyte, we see a splitting of the A_1g_ vibrational mode at 610 cm^–1^, which has not been previously reported. This split can potentially be attributed to the change in symmetry at the boundary layer and due to the electronic effects of the intercalant species, based on similar phenomena observed in graphite systems.^65−67^ Furthermore, it seems likely that the change in observed vibrational modes comes from the reorganization of the multilayered Ti_4_N_3_T_*x*_ MXene structure rather than from intercalated ions. Specifically, due to the multilayered structure of the material, during charge storage under H_2_SO_4_, the out of plane A_1g_ vibrational modes of Ti and N atoms are split into modes adjacent and nonadjacent to the intercalate layer species planes. We hypothesize that this is due to an intercalated layer being created during activation in H_2_SO_4_. Furthermore, due to this information, the mapped spectrum plot (Figure 9b) is able to provide details on the quantity of the MXene surface that has encountered structural modification via ion intercalation. Approximately 25% of the electrode material is observed to have undergone structural reconfiguration for the charge storage mechanism. It seems most likely that H^+^, and not SO_4_^2–^, is intercalated into the Ti_4_N_3_T_*x*_ MXene, as we note that with electrochemical activation under MgSO_4_ electrolyte (Figure 9c,d,g, blue trace), the Raman spectrum of the activated material remains unchanged. Additionally, due to the negative surface charge typically on MXenes, cations are usually the only ions to intercalate between the layers. Further analysis of the MgSO_4_-activated Ti_4_N_3_T_*x*_ material shows that no significant structural changes occurred during the electrochemical experiments. Finally, for the KOH-activated Ti_4_N_3_T_*x*_ (Figure 9e–g, green trace), splitting of the E_1g_ vibrational mode of Ti and N atoms at 254 cm^–1^ and A_1g_ vibrational mode at 426 cm^–1^ are observed, which is in accordance with similar cation intercalation as mentioned above. It is notable that the adjusted spectra show indication of high degrees of intercalation due to the increase in structural modification from the modified E_1g_ and A_1g_ bands. Compared to 25% of the material being modified in the H_2_SO_4_ system, 90% of the mapped spots of the electrode material (Figure 9e,f) are shown to be involved in intercalation, thereby highlighting the compatibility of the KOH electrolyte as observed with the 125% capacitance retention.

### 2.4 Proposed Charge Storage Mechanism

XAS data obtained corroborate that multiple titanium oxidation states (Ti^2+^, Ti^3+^, and Ti^4+^) coexist in Ti_4_N_3_T_*x*_ MXene, with a higher proportion of Ti^4+^ in MXene compared to MAX.^68^ In H_2_SO_4_ electrolyte, the hydronium ions likely assist the redox reactions, resulting in the titanium oxidation state change. Since the termination groups consist of −OH, −O–, and −F, the following pseudocapacitive redox reaction is proposed:
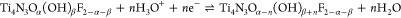
2

This mechanism involves the −O– termination groups being protonated by solvated hydronium ions, which results in the formation of −OH termination groups and vacant Ti sites. The vacant Ti sites are active and would undergo redox reactions in the acidic environment. Due to the multilayered structure of Ti_4_N_3_T_*x*_, the active Ti sites between the layers are accessible by electrolyte ions, leading to interlayer storage and increased capacitance as evidenced by the emergence of a broad, separated redox couple on the CV obtained from the acidic electrolyte.

## 3 Conclusion

We reported the synthesis of multilayered Ti_4_N_3_T_*x*_ MXene by etching the precursor Ti_4_AlN_3_ MAX phase using an oxygen-assisted molten salt fluoride treatment. XRD and SEM analyses indicate etching by a characteristic downshift in the (002) peak and an emergence of multilayered accordion-like morphology in the material, respectively. FTIR spectra suggest a mixture of −F, −O–, and −OH surface termination groups apparent on the MXene. The electrochemical performance of the multilayered Ti_4_N_3_T_*x*_ MXene was characterized in 1 M aqueous H_2_SO_4_, MgSO_4_, and KOH electrolytes. In each electrolyte, electrochemical activation of the material occurred when performing cyclic voltammetry at a 50 mV s^–1^ scan rate over a 10 day period (up to 12000 cycles), leading to capacitance at 2 mV s^–1^ increasing by 300% in H_2_SO_4_, 140% in MgSO_4_, and 500% in KOH. Overall, the capacitance was highest in H_2_SO_4_ before and after activation, achieving 125 F g^–1^ and over 575 F g^–1^, respectively. The capacitance increase in H_2_SO_4_ has been attributed to an interlayer redox charge storage mechanism occurring between the acidic protons and the −O– termination groups. The capacitance increase in MgSO_4_ and KOH has been attributed to a successive ion intercalation pseudocapacitive mechanism from the Mg^2+^ and K^+^ ions, respectively. Moreover, following activation in H_2_SO_4_, changes to the material’s EIS spectra and GCD curves further indicate the presence of Faradaic redox reactions as the dominant charge storage mechanism. Physical characterization of the Ti_4_N_3_T_*x*_ MXene before and after electrochemical characterization also showed maintained structural and morphological integrity of the material in each electrolyte, with some surface oxidation occurring after activation. We believe that the activation period can be reduced through further studies which are currently underway. Moreover, the large operating voltage window of multilayered Ti_4_N_3_T_*x*_ in aqueous environments warrants investigation into the performance of the material in nonaqueous systems and in two-electrode devices where larger operating windows may be achieved.

## 4 Experimental Section/Methods

### 4.1 Material Synthesis

#### 4.1.1 Synthesis of Ti_4_AlN_3_ MAX

The Ti_4_AlN_3_ MAX phase was synthesized by grinding powders of Ti (Sigma-Aldrich, 99.7%, 100 mesh), Al (Sigma-Aldrich, 99%, 30 μm), and TiN (Sigma-Aldrich, 99%, 3 μm) in a 1:1.2:2.05 molar ratio in an agate mortar for 10 min. The mix was then sintered in a tube furnace (CM Furnace Inc. 1730-20 HT) at 1400 °C for 30 h at a ramp rate of 10 °C min^–1^ under a constant Ar flow (Airgas, Ultra High Purity). The resulting Ti_4_AlN_3_ pellet was ground in an agate mortar in preparation for etching.

#### 4.1.2 Synthesis of Molten Salt Treated Ti_4_AlN_3_ (Ti_4_AlN_3_-MST)

The Ti_4_N_3_T_*x*_ MXene was synthesized via selective etching of the Al layer from the Ti_4_AlN_3_ MAX phase powder using an oxygen-assisted molten salt treatment method. The molten salt fluoride (MSF) mixture consisted of KF (Alfa Aesar, 99%), LiF (Alfa Aesar, 325 mesh, 98.5%), and NaF (Alfa Aesar, 99%), in a eutectic mass ratio of 59:29:12 and was mixed with the already synthesized Ti_4_AlN_3_ MAX powder in a 1:1 mass ratio. The combined MAX:MSF mixture was then ground for about 10 min in an agate mortar and transferred into a crucible boat, which was placed in a quartz tube furnace (ATS Series 3210). The furnace was ramped at a rate of 10 °C min^–1^ up to 550 °C and held for 5 h under a constant Ar flow of 360 mL min^–1^. Afterward, the Ar flow was shut off, and the other end of the tube with outlet 3/16 in. ID tubing was opened to air for 1 h to allow for controlled oxygen flow. The furnace was then sealed for 2 h for continued etching of the Al from the MAX:MSF mixture. After this time, the tube furnace was turned off and allowed to cool to room temperature. The Ti_4_AlN_3_-MST was then collected, weighed, and transferred into a vial.

#### 4.1.3 Synthesis of Multilayer (ML) Ti_4_N_3_T_*x*_ MXene

About 0.5 g of the etched Ti_4_AlN_3_-MST was ground and acid-washed by mixing with 20 mL of 4 M formic acid (Sigma-Aldrich, 95%) in a beaker similar to previous synthesis work with Ti_2_NT*_x_*.^50,69^ The beaker contents were stirred for 1 h at 500 rpm using a Teflon-lined stir bar. The resulting solution was then membrane-filtered onto a 0.10 μm polycarbonate membrane (Whatman Nucleopore) and washed continuously by adding deionized water (18.2 MΩ cm, Milli-Q) until a pH of 6 was attained. At the end of the wash cycle, the Ti_4_N_3_T_*x*_ was then collected, dried in a vacuum oven at 40 °C overnight, transferred into a vial, and stored in a glovebox.

### 4.2 Physical Characterization

The bulk crystalline structure of the material was characterized by X-ray diffraction (XRD) using a Rigaku Miniflex 6G X-ray diffractometer equipped with Cu Kα radiation (λ = 0.154 nm). The XRD was operated over a 2θ range of 3° to 70° at a scan rate of 2.0° min^–1^. FTIR was conducted on a Bruker INVENIO-R instrument with a diamond ATR module installed. Physical surface area was determined by N_2_-physisorption (Quantachrome Autosorb-iQ) with the Brunauer–Emmett–Teller (BET) method. The material was degassed in vacuum at 80 °C for 6 h before the measurement. Raman spectroscopy was carried out using a Renishaw inVia Qontor instrument with a 532 nm laser, an 1800 lines/mm grating, and a 50× long objective lens, unless stated otherwise. The morphology of the MXenes was observed with a JSM-IT200 scanning electron microscope (SEM) equipped with energy-dispersive X-ray spectroscopy (EDS). Surface characterizations were performed using X-ray photoelectron spectroscopy (XPS, Omicron XPS system with Argus detector courtesy of TAMU Materials Characterization Facility, RRID:SCR_022202). For survey scans, XPS analysis was done with the CAE as 100 eV and the dwell time as 0.05 s. For high-resolution scans, XPS analysis was done with the CAE as 40 eV and the dwell time as 0.05 s, with three spectra collected to be averaged out for the overall scan. For the X-ray 558 Control, the emission current was set to 15 mA and the anode current to 15 kV, making the X-ray power 225. For the CN10 neutralizer settings, the emission current was set at 10 MA and the beam energy at 2 eV. The aperture was set at 3 or 5, making the aperture coefficients *a* and *b* 304.3 and 0.91 or 39.2 and 0.43, respectively. XAS measurements were performed in fluorescence mode at the multipurpose beamline for spectroscopy, 12-BM, at the Advanced Photon Source (APS) located at Argonne National Laboratory (ANL). A defined beam size of 0.5 × 0.8 mm^2^ using slits and an incident photon flux of ∼10^11^ photons s^–1^ were used. XANES data were collected in the vicinity of the Ti K-edge (4966 eV) at ambient temperature. Ti foil, TiH_2_, TiN, and TiO_2_ rutile were investigated in fluorescence to obtain the reference spectra. XAS data were processed using the Demeter software package with the built-in AUTOBK algorithm used to normalize the absorption coefficient.

### 4.3 Electrode Preparation

Electrodes were prepared via a slurry method with the composition of 85% Ti_4_N_3_T_*x*_ MXene, 10% carbon black (Super P, Alfa Aesar) and 5% polyvinylidene fluoride (PVDF) in *N*-methyl-2-pyrrolidone (NMP). Additional NMP was added to the mixture, until a preferred slurry consistency was achieved. The slurries were then manually painted onto 18 mm diameter conductive carbon paper substrates (5.8 mΩ cm^–1^, MSE Supplies) and dried in a vacuum oven for 8 h at 80 °C. Electrode mass was obtained by subtracting the substrate mass from the total mass after drying. A mass loading of ∼3 mg was used for each electrode.

### 4.4 Electrochemical Cell Setup

The experiment was carried out in a three-electrode setup (PAT Series, EL-Cell) with a Ti_4_N_3_T_*x*_ MXene electrode as the working electrode, activated carbon on stainless steel as a pseudoreference electrode, and a conductive carbon cloth as a counter electrode (1000 m^2^ g^–1^, MSE Supplies). Activated carbon was used as a pseudoreference electrode to simulate performance in an asymmetric two-electrode setup. Titanium foil acted as a single-use current collector for both the working and counter electrodes. Working and counter electrodes were separated using two porous separators (21.6 mm × 0.26 mm) saturated with approximately 250 μL of electrolyte.

### 4.5 Electrochemical Measurements

All electrochemical measurements were performed using a Biologic SP-300 potentiostat. The electrodes were tested in aqueous 1 M H_2_SO_4_, 1 M MgSO_4_, and 1 M KOH solutions. Galvanostatic charge–discharge (GCD) and potentiostatic electrochemical impedance spectroscopy (EIS) measurements were taken in each electrolyte environment before and after the activation process. Before activation, a stable voltage window was determined using CV by expanding the voltage window until the onsets of H_2_ and O_2_ evolution reactions were reached, as indicated by a sharp increase in current magnitude. After the first series of measurements were finished before activation, the voltage window was adjusted in response to the activation process for carrying out subsequent measurements. CV was performed from scan rates of 2 to 1000 mV s^–1^, and GCD was tested from 2 to 100 A g^–1^ both before and after activation. EIS was conducted at open circuit potential using a frequency range from 200 kHz to 10 mHz at an amplitude of 10 mV both before and after activation.

### 4.7 Capacitance Calculations

Gravimetric specific capacitance (F g^–1^) values were calculated using
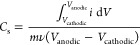
3where *C*_s_ is the gravimetric capacitance, *V*_cathodic_ (V) and *V*_anodic_ (V) represent cathodic and anodic potential boundaries, respectively, *i* (A) represents current, *m* (g) represents electrode mass, and ν (mV s^–1^) represents the scan rate.

## Supporting Information Available

Ti_4_AlN_3_ and Ti_4_N_3_T_*x*_ pore size distrubtions, SEM images with EDS, full FTIR spectra, CV comparison of pristine and activated Ti_4_N_3_T_*x*_, capacities of pristine and activated Ti_4_N_3_T_*x*_ in H_2_SO_4_, MgSO_4_, and KOH, full FTIR spectra of activated Ti_4_N_3_T_*x*_ in each electrolyte, and comparison of reported capacitances and retentions of MXenes (PDF)
