# Supplementary material for: Switchable Charge Storage Mechanism via in Situ Activation of MXene Enables High Capacitance and Stability in Aqueous Electrolytes
Source: ACS Nano. 2024 Feb 19;18(9):7180–91. doi: 10.1021/acsnano.3c12226 (PMC10919077; doi:10.1021/acsnano.3c12226)
Supplement: Supplementary file 1 — nn3c12226_si_001.pdf [file nn3c12226_si_001.pdf]

Supporting Information

Switchable Charge Storage Mechanism via In-Situ Activation of MXene Enables High Capacitance and Stability in Aqueous Electrolytes

*Cheng-Che Hsiao, James Kasten, Denis Johnson, Bright Ngozichukwu, Ray M. S. Yoo, Seungjoo Lee, Ali Erdemir, and Abdoulaye Djire**

C. Hsiao, J. Kasten, D. Johnson, B. Ngozichukwu, R. M. S. Yoo, A. Djire:
Artie McFerrin Department of Chemical Engineering, Texas A&M University, College Station, TX 77843, USA

S. Lee, A. Erdemir:
J. Mike Walker '66 Department of Mechanical Engineering, Texas A&M University, College Station, TX 77843, USA

A. Erdemir, A. Djire:
Department of Materials Science & Engineering, Texas A&M University, College Station, TX 77843, USA

*Corresponding Author: adjire@tamu.edu

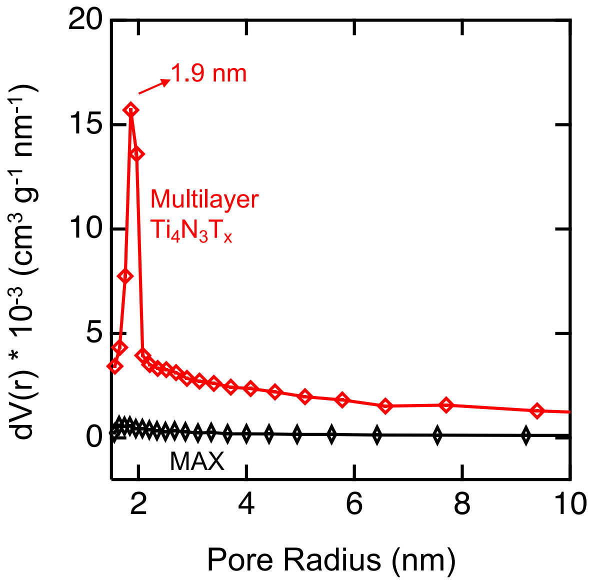

Figure S1. Pore size distribution of MAX and $Ti_4N_3T_x$ MXene.

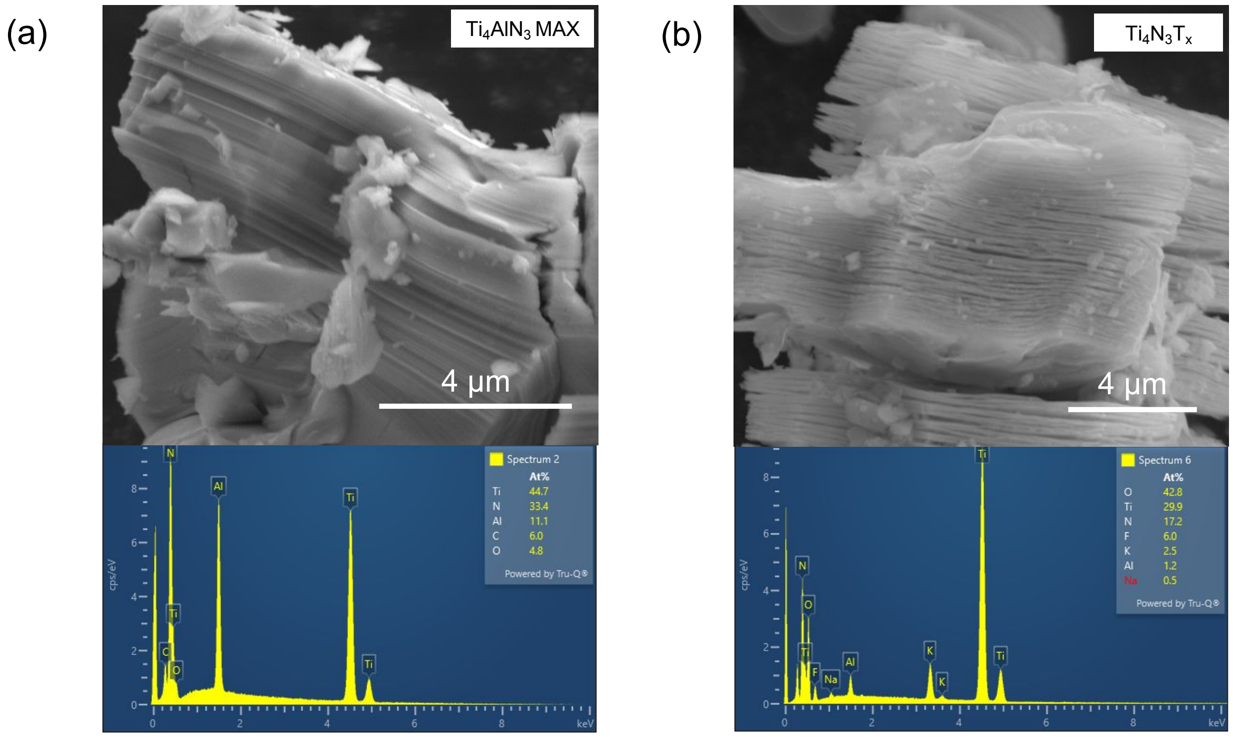

Figure S2. SEM images showing the morphology of (a) Ti_4AlN_3 MAX and (b) $Ti_4N_3T_x$ MXene. And the EDS analysis showing the elemental composition of the material.

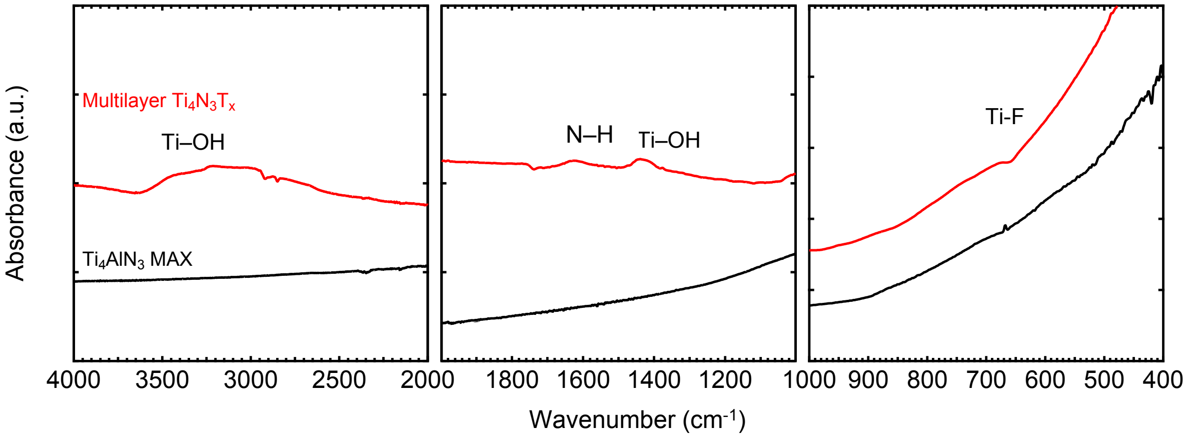

Figure S3. FTIR spectrum for the synthesized Ti_4AlN_3 MAX and multilayer $Ti_4N_3T_x$ MXene.

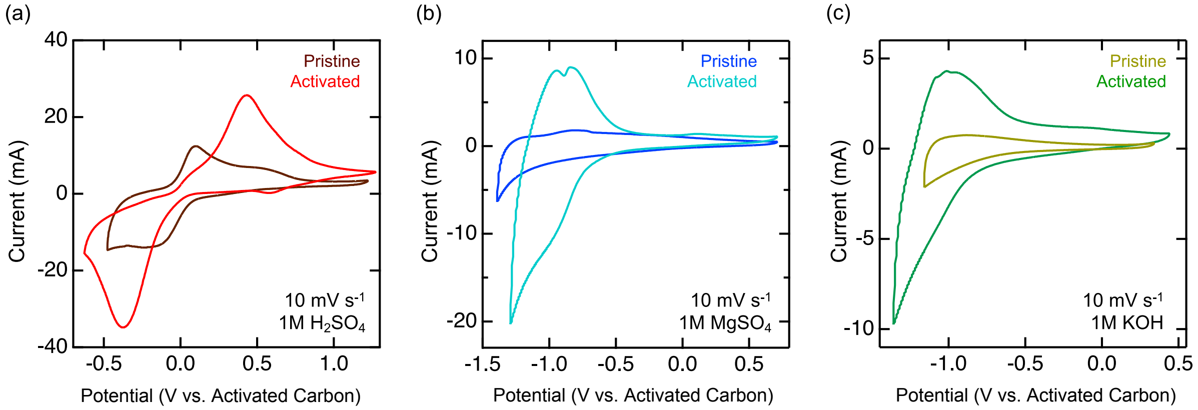

Figure S4. Cyclic voltammogram comparison of pristine and activated electrodes in each electrolyte at 10 mV s^{-1} scan rate: (a) H_2SO_4, (b) $MgSO_4$, (c) KOH.

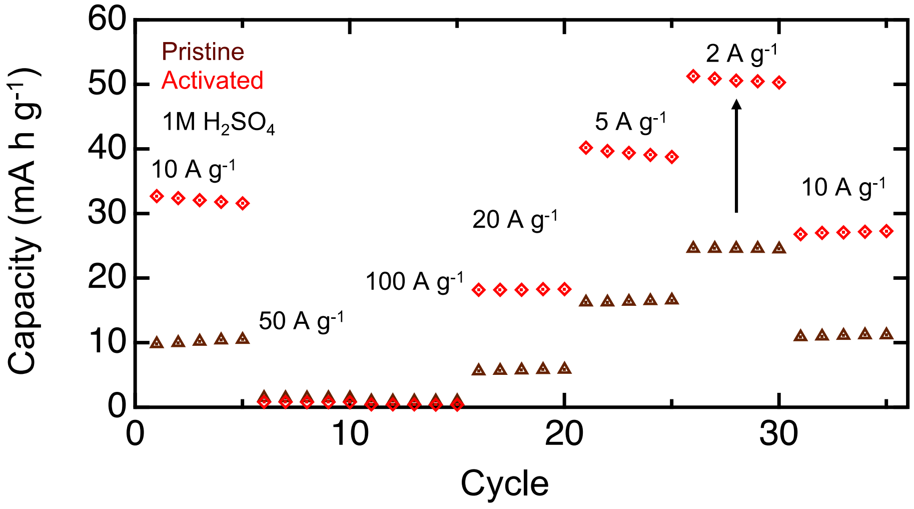

Figure S5. Comparison of the capacity of the $Ti_4N_3T_x$ electrode before and after the activation in 1M H_2SO_4, showing over 100% increase at low discharge rates.

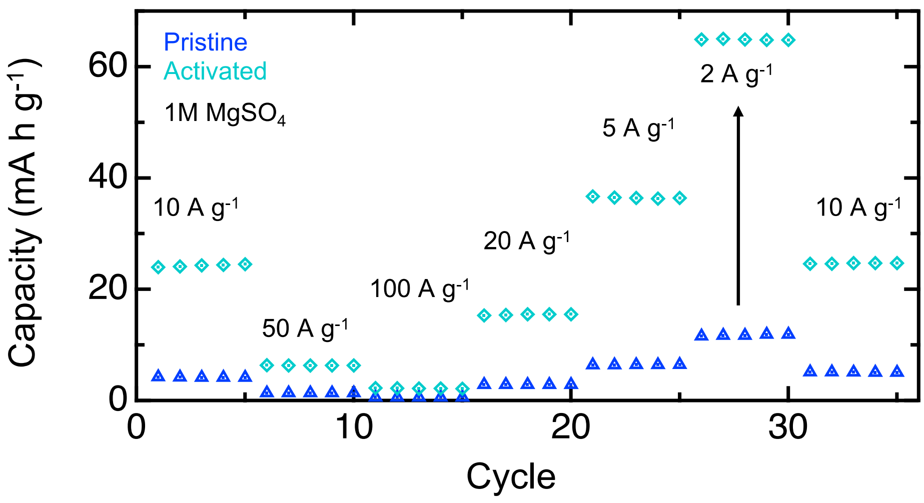

Figure S6. Comparison of the capacity of the pristine and activated $Ti_4N_3T_x$ electrode activation in 1M $MgSO_4$. The activated electrode has 5 times the capacity of the pristine electrode at low discharge rate of 2 A g^{-1}.

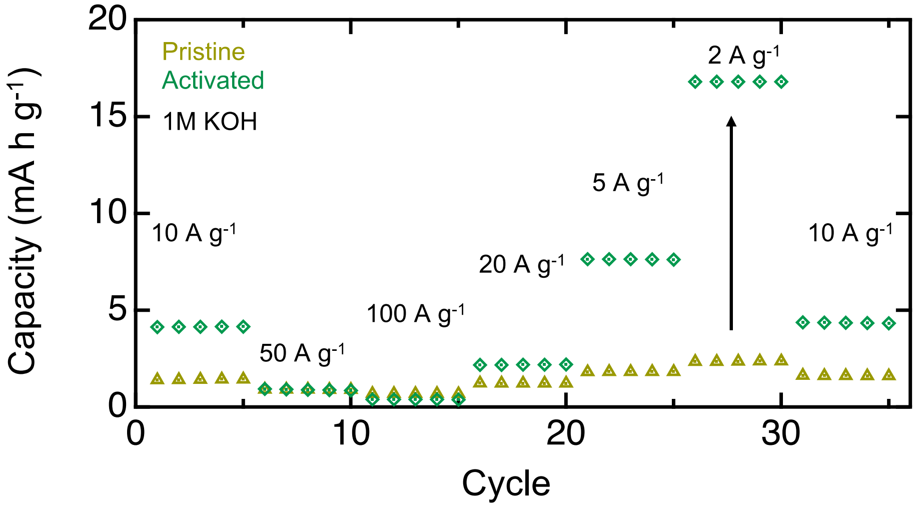

Figure S7. Comparison of the capacity of the $Ti_4N_3T_x$ electrode before and after the activation in 1M KOH. A **600%** increase at low discharge rate is shown.

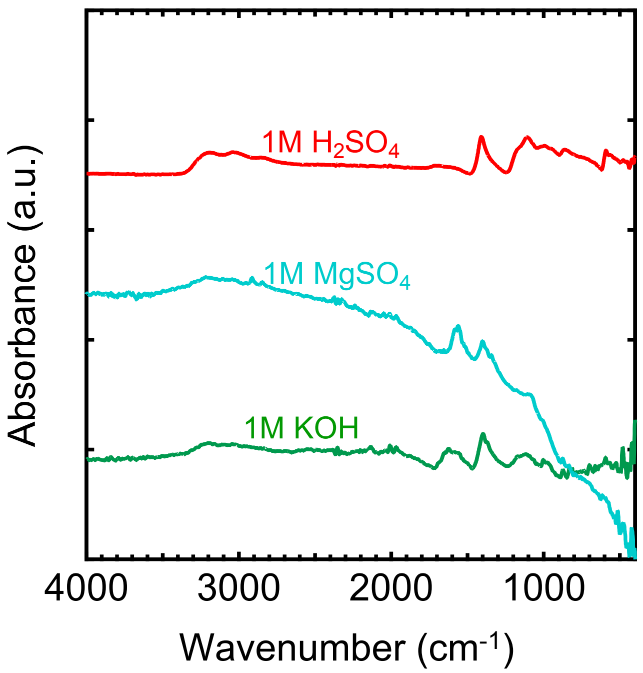

Figure S8. Full FTIR spectrum of post electrochemical experiment characterization for the electrodes in each electrolyte.

Table S1. Reported capacitances and retentions of high performing MXenes in aqueous systems.

MXene	Electrolyte	Max capacitance @ 2 mV s^{-1} (F g^{-1})	Retention	Cycling rate; Number of cycles	Reference
Ti_4N_3 (clay)	1M H_2SO_4<br>1M $MgSO_4$<br>1M KOH	600<br>190<br>150	240%<br>220%<br>125%	50 mV s^{-1}; 50,000<br>50 mV s^{-1}; 11,000<br>50 mV s^{-1}; 12,000	This work
Ti_3C_2 (clay)	1M H_2SO_4<br>1M $MgSO_4$<br>1M KOH	245<br>~95<br>~73	~100%<br>--<br>--	10 A g^{-1}; 10,000	[1, 2]
V_2C (delaminated)	6M KOH	260	121%	10 A g^{-1}; 60,000	[3]
V_4C_3 (clay)	1M H_2SO_4	209	97%	10 A g^{-1}; 10,000	[4]
Mo_2C (delaminated)	1M H_2SO_4	196	~100%	10 A g^{-1}; 10,000	[5]
Nb_4C_3 (delaminated)	1M H_2SO_4<br>1M $MgSO_4$<br>1M KOH	~275<br>~150<br>~205	76%<br>59%<br>49%	2 A g^{-1}; 5,000	[6]
Ti_2N (clay)	1M H_2SO_4<br>1M $MgSO_4$<br>1M KOH	89<br>201<br>41	60%<br>140%<br>110%	5 A g^{-1}; 500<br>5 A g^{-1}; 1,000<br>5 A g^{-1}; 600	[7]

References:

[1] M. Ghidiu, M. R. Lukatskaya, M.-Q. Zhao, Y. Gogotsi, and M. W. Barsoum, "Conductive two-dimensional titanium carbide 'clay' with high volumetric capacitance," *Nature,* vol. 516, no. 7529, pp. 78-81, 2014, doi: 10.1038/nature13970.

[2] M. R. Lukatskaya *et al.*, "Cation Intercalation and High Volumetric Capacitance of Two-Dimensional Titanium Carbide," *Science,* vol. 341, no. 6153, pp. 1502-1505, 2013, doi: doi:10.1126/science.1241488.

[3] T. Zhang, K. Matthews, A. VahidMohammadi, M. Han, and Y. Gogotsi, "Pseudocapacitance of vanadium carbide MXenes in basic and acidic aqueous electrolytes," *ACS Energy Letters,* vol. 7, no. 11, pp. 3864-3870, 2022.

[4] X. Wang *et al.*, "Two-dimensional V4C3 MXene as high performance electrode materials for supercapacitors," *Electrochimica Acta,* vol. 307, pp. 414-421, 2019.

[5] J. Halim *et al.*, "Synthesis and Characterization of 2D Molybdenum Carbide (MXene)," *Advanced Functional Materials,* vol. 26, no. 18, pp. 3118-3127, 2016, doi: https://doi.org/10.1002/adfm.201505328.

[6] S. Zhao *et al.*, "Flexible Nb4C3Tx Film with Large Interlayer Spacing for High-Performance Supercapacitors," *Advanced Functional Materials,* vol. 30, no. 47, p. 2000815, 2020, doi: https://doi.org/10.1002/adfm.202000815.

[7] A. Djire, A. Bos, J. Liu, H. Zhang, E. M. Miller, and N. R. Neale, "Pseudocapacitive storage in nanolayered Ti2NT x MXene using Mg-ion electrolyte," *ACS Applied Nano Materials,* vol. 2, no. 5, pp. 2785-2795, 2019.
